# Advances and challenges in CRISPR-based real-time imaging of dynamic genome organization

**DOI:** 10.3389/fmolb.2023.1173545

**Published:** 2023-03-31

**Authors:** Jenna Thuma, Yu-Chieh Chung, Li-Chun Tu

**Affiliations:** ^1^ Department of Biological Chemistry and Pharmacology, The Ohio State University, Columbus, OH, United States; ^2^ Interdisciplinary Biophysics Graduate Program, The Ohio State University, Columbus, OH, United States; ^3^ Center for RNA Biology, The Ohio State University, Columbus, OH, United States; ^4^ The Ohio State University Comprehensive Cancer Center, The Ohio State University, Columbus, OH, United States

**Keywords:** chromatin dynamics, chromosome conformation, genome organization, CRISPR, live-cell imaging, single-particle tracking

## Abstract

Nuclear chromosome compaction is non-random and dynamic. The spatial distance among genomic elements instantly modulates transcription. Visualization of the genome organization in the cell nucleus is essential to understand nuclear function. In addition to cell type-dependent organization, high-resolution 3D imaging shows heterogeneous compaction of chromatin organization among the same cell type. Questions remain to be answered if these structural variations were the snapshots of dynamic organization at different time points and if they are functionally different. Live-cell imaging has provided unique insights into dynamic genome organization at short (milliseconds) and long (hours) time scales. The recent development of CRISPR-based imaging opened windows for studying dynamic chromatin organization in single cells in real time. Here we highlight these CRISPR-based imaging techniques and discuss their advances and challenges as a powerful live-cell imaging method that poses high potential to generate paradigm-shifting discoveries and reveal functional implications of dynamic chromatin organization.

## 1 Introduction

Genetic information coded by DNA is highly regulated to express cell type-specific genes. To achieve this regulation, DNA dynamically associates with proteins forming a complex known as chromatin. Chromatin is hierarchically folded to maintain homeostasis and allow rapid response to stimuli (Schoenfelder and Fraser, 2019; Boopathi et al., 2020; Davidson and Peters, 2021). To fully understand the regulation of genome function, scientists must be able to manipulate chromatin *in vivo* and detect the real-time changes in single cells. Traditional methods used to study chromatin organization have been largely “snapshot”-based, while these methods have advanced our knowledge of genome architecture, our understanding of dynamics and heterogeneity across a cell population is still in its infancy. Elucidating the temporal genome organization is key for understanding genome function. Direct visualization of chromatin dynamics endogenously in living cells is essential to advance our understanding of chromatin movement, structure, and function.

### 1.1 Primary Tools for investigating genome organization before CRISPR

DNA dyes that interact with the DNA double helix allow visualization at the whole genome level (Kapuscinski, 1995; Stockert et al., 2014). Although scientists were able to distinguish mitotic and interphase chromosomes, they were unable to study the details of genome organization. The development of fluorescence *in-situ* hybridization (FISH) (Levsky and Singer, 2003) and chromosome conformation capture techniques (Dekker et al., 2013) enabled genome research in fixed cells at high resolution. Using these techniques, scientists characterized the mechanisms governing chromatin loop formation, identified topologically associating domains (TADs) (Davidson and Peters, 2021), visualized X-chromosome inactivation (Chaumeil et al., 2004), and, more recently, simultaneously imaged genome and spatial transcriptomics [for reviews (Zhuang, 2021; Moses and Pachter, 2022)]. Despite having made breakthrough discoveries on genome organization, sample preparation in these techniques requires invasive fixation and denaturation which poses a risk of introducing artifacts. Fixation reagents were reported to reduce the nuclear size (Amiad-Pavlov et al., 2021) and change the nuclear environment (Irgen-Gioro et al., 2022). Data discrepancies from technologies were reported (Finn et al., 2019), reflecting the heterogeneity of genome organization and methodological limitations in different technologies.

## 2 CRISPR as a versatile tool for genome studies in living cells

Tracking dynamic genome organization in single living cells at high resolution allows us to analyze the temporal and cell-to-cell variation of genome organization separately, providing a complete understanding of the genome function. In 2020, the Nobel Prize in Chemistry was awarded to the discovery of clustered regularly interspaced short palindromic repeats (CRISPR) as one of the most influential breakthroughs and powerful techniques for precise genome editing of the 21st century (Ledford and Callaway, 2020). Beyond genome editing, CRISPR has been repurposed for genome imaging, epigenome engineering, biosensing (Safdar et al., 2022), and other areas in various biological systems [for a complete review, see (Wang et al., 2016; Knott and Doudna, 2018)]. The myriad applications of CRISPR technologies take advantage of the precise DNA targeting of CRISPR systems to modify, manipulate, or detect the genome *in vivo*. Deactivating the CRISPR-associated (Cas) proteins converts a CRISPR system from a genome editing tool to an imaging tool. In the type-II CRISPR/Cas9 system from *Streptococcus pyogenes*, the two substitution mutations, D10A and H840A, deactivated Cas9 to the nuclease-dead Cas9 (dCas9) which binds to DNA but does not generate double strand breaks (DSBs) (Jiang et al., 2015). This system includes four important elements - CRISPR RNA (crRNA), *trans*-activating crRNA (tracrRNA), Cas protein, and protospacer adjacent motifs (PAMs). While the crRNA guides the Cas protein to the target by base pairing, the tracrRNA serves as the glue that holds crRNA, and Cas together. Genetic fusion of the crRNA and tracrRNA creates single guide RNAs (sgRNAs); this is a common strategy to simplify the CRISPR systems (Chen et al., 2013). The PAM, usually 2–6 base pairs long at the targeting site, is required for the initial recognition by Cas proteins.

### 2.1 CRISPR-Cas9-GFP and CRISPR-Cas variants

**FIGURE 1:**
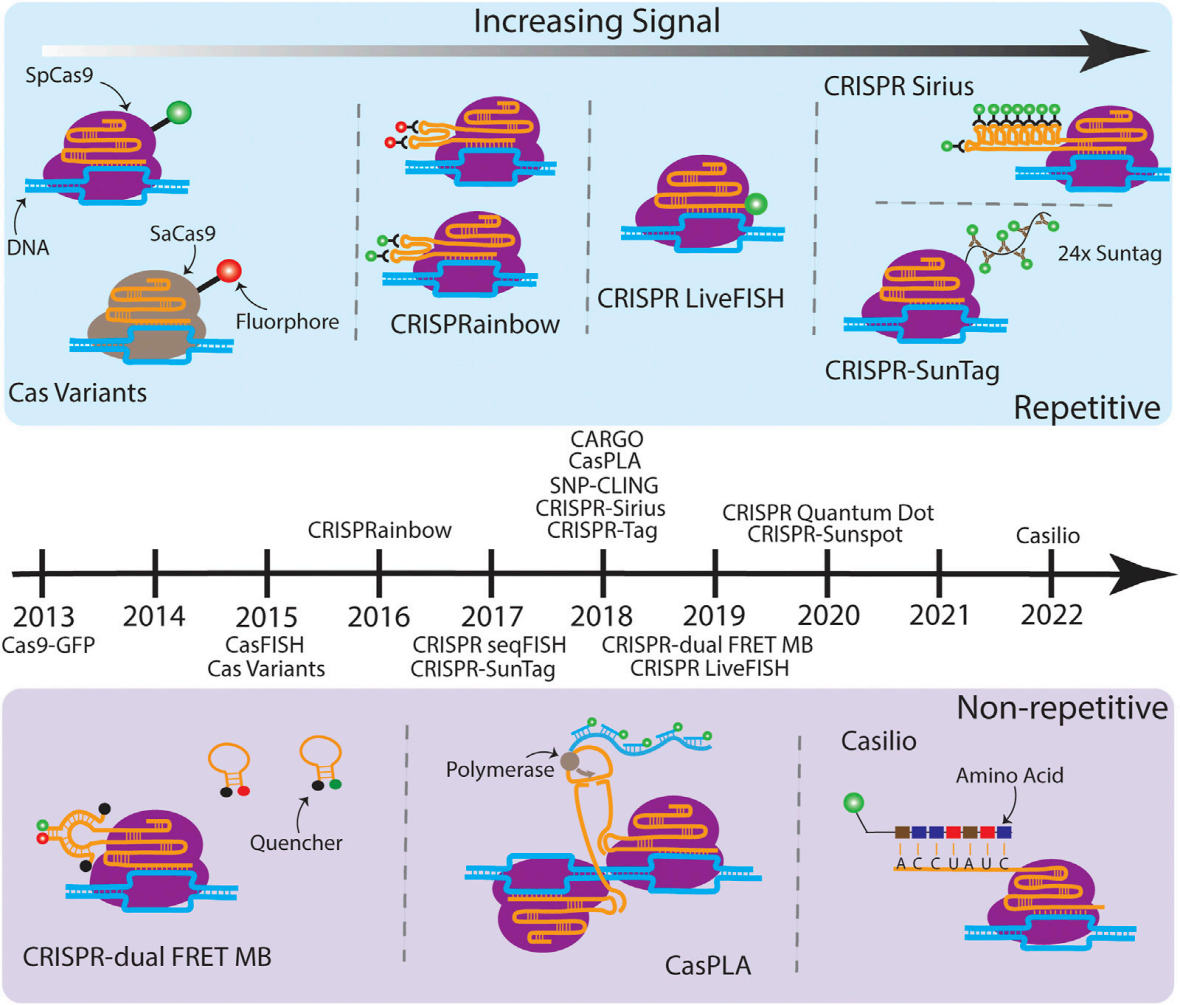
A timeline of CRISPR-based imaging techniques. (Above the timeline) Various techniques which target repetitive sequences are illustrated moving from less SNR (left) to higher SNR (right). Cas variants consist of Cas proteins from orthogonal species fused to different fluorophores, which enable simultaneous multicolor imaging on multiple genomic loci (Chen et al., 2016). CRISPRainbow introduces ×2 stem loops, MS2, BoxB, and PP7, from bacteriophages into the sgRNA scaffold, which then bind with high affinity to their corresponding coat proteins with fluorescent tags, also enabling multicolor imaging of up to seven distinct repetitive sequences (Ma et al., 2016a). CRISPR LiveFISH attached a synthetic dye to the 5′ end of the sgRNA (Wang et al., 2019). CRISPR-Sirius builds on CRISPRainbow by expanding the MS2 and PP7 stem loops to ×8, increasing the fluorescent output and allowing tagging of lowly repetitive sequences (Ma et al., 2018). CRISPR-SunTag fused an array of ×24 GCN4 sites to the dCas9, which recruits the scFv antibody tethered to a green fluorescent protein. (Below the timeline) Various techniques which target non-repetitive sequences are illustrated. CRISPR-dual FRET MB engineered a gRNA which has a stem loop complementary to a set of molecular beacons containing a FRET pair. The donor and acceptor MB alone interact with their quenchers and are non-fluorescent (Mao et al., 2019). Once bound, the FRET pairs are in close proximity to one another, resulting in a bright fluorescent output. CasPLA is designed to detect single-nucleotide variations (SNVs) by using a pair of Cas9 complexes which target adjacent sequences (Zhang et al., 2018). The successful targeting leads to the ligation of linear probes bound to each sgRNA, which starts rolling circle amplification by the DNA polymerase. The generated long repetitive tail can be bound by fluorescent oligonucleotide probes for imaging. In the presence of a SNV, one of the Cas9 complexes will not bind which results in no fluorescent signal. Finally, Casilio amplifies signal by creating repeats within the sgRNA which are then bound by a fluorescently tagged RNA binding protein named Pumilio (Clow et al., 2022). Techniques listed on the timeline but not illustrated due to the space limit are: Cas9-GFP (Chen et al., 2013), CasFISH (Deng et al., 2015), CRISPR seqFISH (Takei et al., 2017), SNP-CLING (Maass et al., 2018), CRISPR-Tag (Chen et al., 2018), CRISPR Quantum Dot (Yang et al., 2020), and CRISPR-Sunspot (Sun et al., 2020).

The first CRISPR imaging used a GFP fused dCas9 (dCas9-GFP) to label specific genomic loci as a monochromatic system (Chen et al., 2013) (Figure 1). CRISPR-dCas9-GFP was demonstrated to label and track repetitive genomic sequences, such as telomeres. For imaging a non-repetitive locus, signal amplification by targeting adjacent sites with a sgRNA cocktail containing 26–72 sgRNAs was necessary. To investigate functional relevance of multiple genomic elements, multicolor CRISPR imaging was developed by using a combination of CRISPR-dCas9 orthologs, such as *Streptococcus pyogenes* (Sp), *Neisseria meningitidis* (Nm), and *Streptococcus thermophilus* (St1) CRISPR-dCas systems for a three-color imaging system (Ma et al., 2015), or *Sp*dCas9 with *Staphylococcus aureus* (Sa) dCas9 for a two-color system (Chen et al., 2016). More recently, Sun and colleagues demonstrated that cellular DNA and mRNA can be visualized simultaneously in single cells by combining fluorescent CRISPR-dCas9 and CRISPR-dCas13 systems (Sun et al., 2020). Using CRISPR variants, although practically available, requires efforts for optimizing the delivery of orthogonal CRISPR-dCas systems and for targeting sites next to different PAMs.

### 2.2 CASFISH and GOLD FISH

CASFISH, the earliest application of CRISPR in a FISH setting, has allowed FISH experiments to be conducted without global denaturation of chromatin by high temperature as in conventional FISH experiments (Deng et al., 2015). Similarly, GOLD FISH was used to label non-repetitive genomic sequences and in chromosome X painting (Wang et al., 2021). In GOLD FISH, a CRISPR-Cas9 nickase and dsDNA helicase, Rep-X, were used to create a single-strand break and locally unwind DNA for hybridizing fluorescent oligos under physiological temperature, minimizing the structural perturbation for chromatin.

### 2.3 CRISPRainbow

The breakthrough of CRISPRainbow is the color expansion (up to seven colors simultaneously using one CRISPR-dCas system) (Ma et al., 2016a). CRISPRainbow makes labeling different chromosomes significantly easier because only one dCas protein is needed and all the sgRNAs for targeting different loci can be cloned into the same plasmid. To establish a broad spectral range, sgRNAs were engineered to include combinations of three RNA hairpins—MS2, PP7, and boxB—each recognized by a distinct bacteriophage RNA coat protein fused to spectrally-distinct fluorescent proteins, MCP-BFP (blue), PCP-GFP (green), and N22-mCherry (red). Additional colors—cyan, yellow, magenta, and white—were generated by the spectral overlap of two or three primary colors. The application of CRISPRainbow was limited to highly repetitive sequences (> a hundred copies). Due to this constraint, only ∼30 genomic loci in the human genome can be labeled by CRISPRainbow.

### 2.4 Extended sgRNA for signal amplification

Signal amplification of CRISPR-based imaging techniques has allowed scientists to track low- or non-repetitive genomic regions with fewer sgRNAs (e.g., 1–4 sgRNAs per locus); this improves the labeling efficiency and expands the applications of the technology. A common strategy to improve the fluorescence signal is to use an array of tandem repeats of fluorophores. Imaging low- and non-repetitive genomic loci was reported by inserting tandem ×14 MS2 stem loops at the 3′ end of the sgRNA (Qin et al., 2017). However, confirmed by real-time PCR analysis, the insertion of highly structured RNA hairpins at 3′ end of the sgRNA promotes degradation in human cells that results in a small cellular sgRNA pool, creating inefficient labeling (Ma et al., 2018). Thermostable octets of MS2 and PP7 aptamers were designed to form three-way junction in the CRISPR-Sirius sgRNA scaffold. This design promotes the stability of CRISPR-Sirius sgRNAs and results in ∼15-fold higher sgRNA concentration in human cells compared to the sgRNA concentration of the 3′ end 14xMS2 array. RNA aptamers, such as broccoli, that directly interact with small synthesized fluorophores, were used to track sgRNA dynamics in living cells (Ma et al., 2016b). However, the fast photobleaching and insufficient brightness of the cell-permeable fluorophore DFHBI-1T limited its application to bulk experiments. Recently, CRISPR-dCas9 combined with U-rich internal loop (URIL) and fluorescent bifacial peptide nucleic acids (fbPNA) was shown to be a potential future labeling strategy for CRISPR-based imaging (Liang et al., 2022).

### 2.5 Live FISH

Instead of tagging sgRNAs with fluorescent protein fused RNA coat proteins (e.g., PCP-GFP etc.), fluorogenic sgRNAs can be made by directly tagging small commercial dyes to the 5’ end of the sgRNA, such as Alexa488-sgRNA (Wang et al., 2019). These synthesized fluorogenic sgRNAs were demonstrated to efficiently label specific loci with repetitive sequences on Chr3 and Chr13 using electroporation in human cells. This approach, termed Live FISH, significantly increased labeling efficiency of genomic loci compared transfected dCas9 and sgRNA plasmids. However, Live FISH labeling was only demonstrated on highly repetitive genomic regions (repeats >350) thus the labeling efficiency of Live FISH on lowly repetitive or non-repetitive genomic regions remains elusive.

### 2.6 CRISPR-Cas9 SunTag and CRISPR-Tag

The dCas9-SunTag system has been used to visualize genomic loci with 21 or 15 copies of repeats [(Ye et al., 2017), For information about SunTag, refer to (Tanenbaum et al., 2014)]. In dCas9-SunTag, the dCas9 was genetically linked to 24 copies of the GCN4 peptide (dCas9-24xGCN4), which serves as binding ligand for the fluorescent protein fused single-chain variable fragment antibody (e.g., scFv-mNeonGreen). Another approach simply integrated an array of 14xGFP to the C-terminus of dCas9 (dCas9-14xGFP), known as CRISPR-Tag (Chen et al., 2018). CRISPR-Tag has increased the signal-to-noise ratio (SNR) threefold compared to the SNR in the single GFP fused dCas9 system (dCas9-GFP) and has successfully labeled a six repeat genomic locus using only four sgRNAs in *C. elegans*.

### 2.7 CRISPR-Casilio

CRISPR-Casilio amplifies the fluorescence signal of sgRNAs by fusing unique repeats of Pumilio/FBF (PUF)-binding sites (PBS) to 3′ end of the sgRNA (sgRNA-×20 PBSc) (Cheng et al., 2016). Pumilio interacts with RNA at specific sequences. Fusing Pumilio with fluorescent proteins produces a fluorescent effector (e.g., Clover-PUF) that labels sgRNA-×20 PBSc. This approach was first used to label telomeres and centromeres in 2016 and, later, to visualize non-repetitive genomic regions using one sgRNA in 2022. CRISPR-Casilio is programmable and therefore allows multicolor imaging on different chromosomal regions. However, the sgRNAs fused with ×20 PBSc were reported to generate many non-specific foci in the cell nucleus in the absence of dCas9 (Hong et al., 2018). The newer version of CRISPR-Casilio, containing ×15 PBSc per sgRNA, addressed the signal specificity issue by re-designing the PBS RNA array and the amino acid sequence of PUF RNA binding domain (Clow et al., 2022).

### 2.8 CRISPR-dual FRET MB

Combining molecular beacons (MBs) as a fluorescence resonance energy transfer (FRET) pair with the CRISPR-dCas9 system have allowed visualization of non-repetitive DNA sequences in living cells. CRISPR/dual-FRET MB used two distinct molecular beacons as a FRET donor and acceptor pair to minimize the noise background of non-specific interacting events in cells since the FRET signals will only be detected when the FRET pairs are in proximity and released from their quenchers. As few as three sgRNAs is sufficient to clearly visualize the non-repetitive *MUC4* locus (Mao et al., 2019).

### 2.9 CasPLA

Precisely detecting single-nucleotide variations (SNVs) helps to understand aging-related diseases. Zhang and colleagues designed CRISPR/Cas9-mediated proximity ligation assay (CasPLA), allowing SNVs to be visualized in single cells (Zhang et al., 2018). CasPLA used a pair of CRISPR-Cas9 complexes to target a non-repetitive mitochondrial DNA (mtDNA) locus. Once stable targeting formed on the two nearby DNA sequences by the pair of CRISPR-Cas9 complexes, the added linear oligonucleotides were then ligated to form a circular DNA, which triggers rolling circle amplification (RCA) to generate additional DNA for signal amplification through hybridization of synthetic fluorogenic oligonucleotides. SNVs in the targeting sequence leads to unsuccessful CRISPR-Cas9 complex base pairing to the target sequence, which fails the formation of a circular DNA and significantly reduces the fluorescence signals. The detection efficiency of active and inactive Cas9 was compared in CasPLA. A slightly higher (∼1.2 fold) efficiency was detected when the dCas9 was used. Although CasPLA is limited to *in vitro* applications, it only needs two CRISPR-Cas complexes to detect one specific non-repetitive sequence. The future development of CasPLA for live-cell imaging will be a great advance of this field.

## 3 Characterizing the real-time dynamics of genomic loci by CRISPR-based imaging techniques

CRISPR-based imaging techniques have been employed to track the movement of specific genomic loci and paint large chromosomal domains in different types of cells. As a highly repetitive sequence (usually >100 copies of repeats), telomeres have been successfully visualized in human osteosarcoma cells (U2OS), human retinal pigment epithelium (RPE), HeLa, HEK293T, plant, mouse epiblast-like (mEpiLCs), and mouse embryonic stem (mESCs) cells by CRISPR-imaging techniques (Chen et al., 2013; Ma et al., 2015; Dreissig et al., 2017; Ye et al., 2017; Gu et al., 2018; Ku et al., 2022). However, thousands of additional repetitive sequences for CRISPR-*sp*dCas9 targeting have been bioinformatically predicted in human cells (Qin et al., 2017; Ma et al., 2018). The comparison of genomic locus mobility along a single chromosome by CRISPR-Sirius showed that loci in silenced or intergenic regions moved subdiffusively at similar speeds but loci on pericentromeric regions moved significantly slower (Ma et al., 2019; Chung et al., 2023). Interestingly, global transcription perturbations by small inhibitors, such as 5,6-Dichloro-1-β-D-ribofuranosyl- benzimidazole (DRB) and flavopiridol, elevated the mobility of telomeres except for actinomycin D, which is known to intercalate into DNA (Ku et al., 2022). Similarly, Chung and colleagues observed elevated mobility on the medium active locus *ZNF358* (TPM (transcription per million) = 37.2) treated with DRB, but not on the silenced gene locus *CYP4F12* (TPM = 0). In mouse embryonic cells (mESC), Gu and colleagues observed increased mobility on the loci with transcription activation using CARGO (chimeric array of gRNA oligonucleotides). Of note, increased genomic locus and chromatin domain dynamics were also observed by single-nucleosome tracking in HeLa cells and by insertion of *LacO* as the labeling site in fibroblasts (Chubb et al., 2002; Nozaki et al., 2017). Ultimately, different observations on genomic locus mobility could simply reflect locus-dependent dynamics.

## 4 Translocation and compaction of large chromosomal domains detected by CRISPR-based imaging techniques

Wang and colleagues tracked chromosomal translocations using LiveFISH (Wang et al., 2019). The two distinct chromosomal regions on chromosome 3 and 13 joined and remained together for hours after active CRISPR RNPs were delivered to create DSBs. Painting large chromosomal domains or entire chromosomes by CRISPR was demonstrated on chromosome 9 and 19 independently using 1 to 802 sgRNAs (Zhou et al., 2017; Feng et al., 2020). Contrary to the static property predicted by simulations (Florescu et al., 2016), chromosome conformations of large chromosomal domains are dynamic and can switch between different conformations by manipulating the epigenetic marks (Feng et al., 2020). As a result, studies which report a single static chromosome conformation for each chromosome do not show the complete picture of dynamic chromosome conformations and functions. Future studies are required to understand if different chromosome conformations correlate to different transcription profiles.

Chromosomal compaction can be quantitatively described by the compaction exponent, the scaling exponent of a power-law relation between genomic distance and spatial distance (Tark-Dame et al., 2011). This method has been used by FISH chromatin tracing and multiplexed imaging groups to characterize polycomb-repressed domains, X chromosome inactivation, and lamina-induced chromosomal stretching in fixed cells (Boettiger et al., 2016; Wang et al., 2016; Sawh et al., 2020). Using this method, CRISPR-based imaging revealed cell cycle-dependent compaction in interphase (Chung et al., 2023). The chromosome 19 long arm packed tighter in early G1 phase compared to the compaction in late G1. This is consistent with the observation detected by chromosome conformation capture techniques, that establishing topologically associating domains (TADs) and compartments takes hours after cells are released from prometaphase arrest (Abramo et al., 2019). This result challenges the long-standing hypothesis of unchanged chromosome compaction throughout the interphase. In the future, we expect the power-law function will provide more quantitative insights into compactions among chromosomal domains in different states in living cells.

## 5 Distinct modes of chromatin dynamics

**FIGURE 2:**
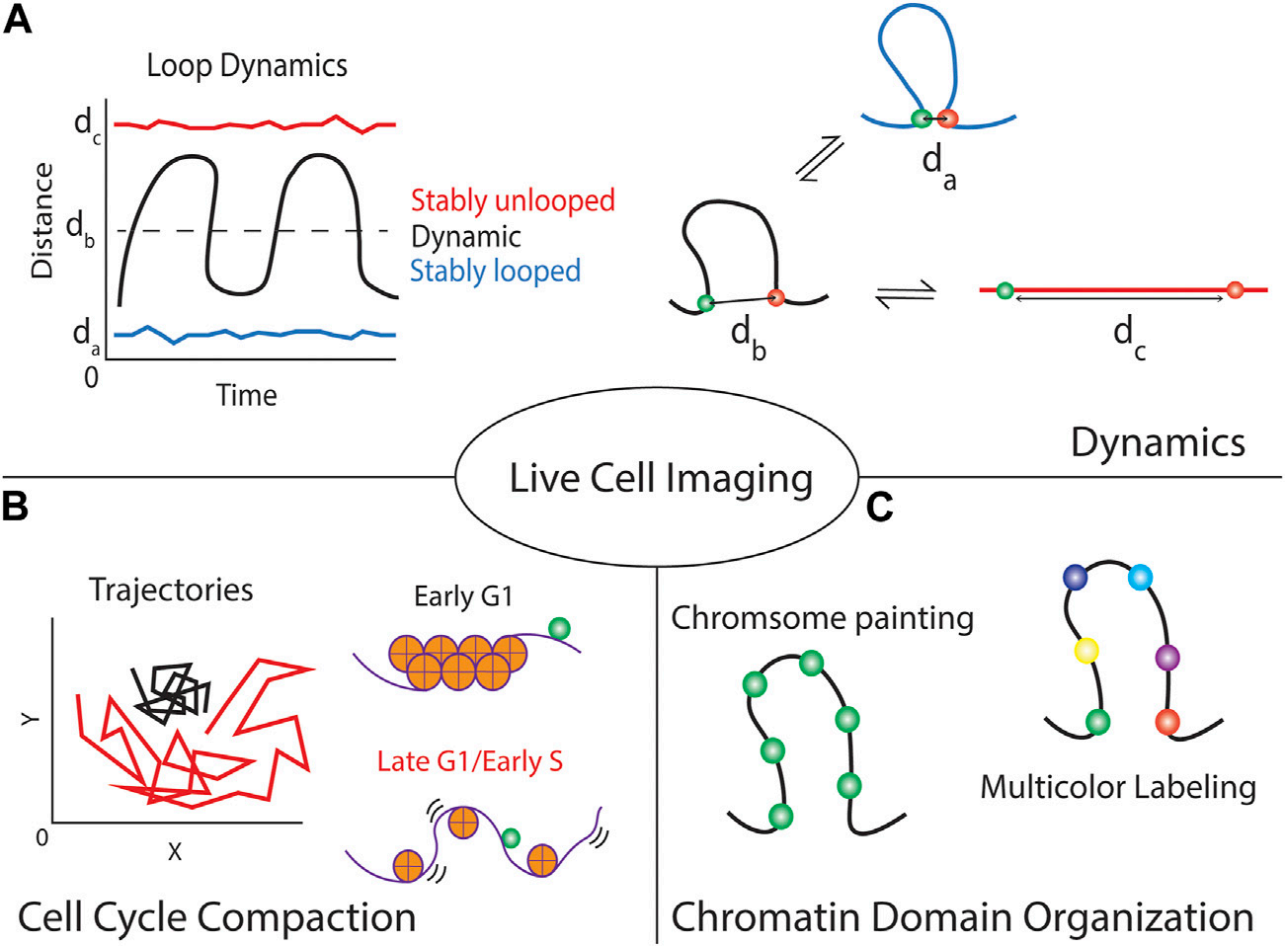
Live-cell CRISPR imaging of genomic loci provides multiple forms of information about the locus and local chromatin domain. **(A)** Focusing on single cells can give temporal dynamics about the local chromatin region, using dual-colored techniques can show if the region is stably looped or unlooped by maintaining a small or large spatial distance between the foci, respectively. Alternatively, it informs if the loop is continuously modulating its structure by large fluctuations in the distance over time. **(B)** These techniques can indicate the local chromatin compaction by tracking the trajectories of the fluorescent foci over time. Highly packed chromatin (e.g., chromatin in early G1 phase) is generally more stiff thus minimizing locus movement compared to loosely packed chromatin (e.g., chromatin in late G1 or early S phase). **(C)** Although introducing multiple labels per chromosomal domain allows the visualization of the chromosome territory (chromosome painting), using a multicolor labeling strategy will provide the structural information in detail (multicolor labeling).

Live-cell imaging of two or more specific genomic loci simultaneously labeled on a single chromosome was used to quantitatively study dynamic chromatin domains and folding (Ma et al., 2019). The spatial distance between locus pairs can inform the folding states within the chromosomal domains. For example, small spatial distances between two labeled loci within a chromosomal domain imply possible loop formation or highly compacted structure. Moreover, the mobility of the loci can be used as an indicator for the stability of the chromatin structure. If the spatial distance of a locus pair stays similar over time (Figure 2A, blue line), the local chromatin structure remains stable and unchanged. An example of such stability was reported for genomic loci measured in early G1 phase (Ma et al., 2019). Highly restricted co-movement of two loci in early G1 phase was detected compared to the movement of the same loci in late G1 or early S phase (Figure 2B). On the other hand, if the spatial distance between a locus pair with a chromosomal domain largely fluctuated over time, the local chromosomal domain is less packed and undergoing dynamic folding (e.g., compaction-relaxtion cycles) (Figure 2A, black line). Another extreme is that the spatial distance between two loci remains long and stays similar (Figure 2A, red line), which suggests stably relaxed chromatin fibers. The chromatin rigidity or attachment of the chromatin to nuclear organelles would be essential to maintain such highly relaxed chromatin structure. Multiple labeled loci (>2 loci) are required to measure the dynamic folding of larger chromosomal domains in sufficient detail (Figure 2C), which emphasizes the importance of robust live-cell multicolor imaging or chromosome paint techniques for future studies.

## 6 Challenges and future perspectives

We have discussed different CRISPR-based imaging techniques that have been improved significantly in brightness and ease of use in the past few years. However, difficulties remain for labs to adopt these techniques. Here we summarize limitations and disadvantages of CRISPR-based imaging techniques that could be further improved in the future: 1) a sgRNA can have different labeling efficiency in different cell types. Optimizations are necessary for each research project. 2) Lentiviral transduction is often used for generating stable cell lines with consistent locus signals. However, lentiviral transduction is more labor-intense, compared to transfection, and the efficiency depends on quality of the viral particles. 3) CRISPR-based imaging techniques have not yet been made for high-throughput applications. 4) High sensitivity of the microscope system is required to detect weak fluorescence signals at low noise background for imaging low- and non-repetitive sequences. Further improvements in CRISPR-dCas delivery, sgRNA design, fluorophore size, and microscopy will expand the applications and facilitate the adoption of these techniques. Nevertheless, CRISPR-based imaging techniques are not replaceable by other imaging techniques. Studying spatio-temporal dynamics of genome organization non-invasively under physiological conditions and endogenous sequences in single chromosomes in single cells cannot be achieved by approaches using fixed cells or whole genome labeling methods.
